# Treatment of Pulpless Teeth

**Published:** 1883-10

**Authors:** W. O. Barrett


ARTICLE VII.

### ON THE TREATMENT OF PULPLESS TEETH.

CASE I.

We will suppose a case: Our patient has had her front teeth filled some time, and with the exception of slight sensitiveness to thermal changes they have been comfortable. But recently one of them became sore, felt longer than its fellows, and finally the face commenced swelling. Perhaps our patient applies at this time, or more likely she applies sooner, when the diagnosis is more difficult. It is not with the diagnosis we propose to deal; for generally this is not hard to make out; but the stage at which the case is first seen will greatly modify the treatment, therefore we will assume that our first patient applies to us in the early stages. We find the tooth quite sore to the touch, and generally slightly discolored. We drill into the pulp chamber with a spear drill, and our diagnosis is verified by the painless plunge of the instrument into the pulpless canal. Having reamed out the opening with a fissure burr, so as to gain easy access, we next proceed to explore the canal with a small nerve-broach, in order to bring away any shreds of pulp not yet fully disintegrated. This done we wash out gently with warm water, and if bleeding has followed the use of the broach we keep up a gentle washing until it ceases. Then, wrapping a shred of cotton upon a Donnelson broach we dip in alcohol and wipe out the canal, repeating until it is absolutely clean; at the last, leaving the cotton in the canal and dismissing the patient until another day when we shall most likely find the soreness has subsided sufficiently to admit of filling the tooth.

CASE 2.

If, however, suppuration has set in before we see the case, we shall most likely have the discharge of a small quantity of pus upon the withdrawal of the drill, in which event, it is our custom to dismiss the patient without making further application, leaving the canal unclosed, that all the pus may drain off. We do not like to leave the canal unstopped longer than a few hours at a time, as food will crowd in and stimulate the decomposing pulp as a cause of offense. We therefore instruct our patient to call on the following day when, instead of using the simple alcohol, we wipe out the canal with a mixture composed of equal parts of wood creosote and the simple tincture of iodine.

Why do we use these agents ?

Because we know we have in this tooth decomposed and decomposing organic matter. The iodine deals with the already decomposed matter as a disinfectant, whilst the creosote deals with the undecomposed and decomposing matters as an antiseptic.

Iodine acts chemically by the destruction of the foul sulphuretted hydrogen. Seizing its hydrogen hydriodic acid is formed, and sulphur precipitated. Then the alcoholic vehicle of the tincture has an antiseptic effect. Creosote acts by holding in check that series of retrogressive chemical changes by which poisonous gases are formed.

In the treatment of this tooth we need both these agents. Yes, all three ; the alcohol of the tincture doubtless plays an important part. We use great care in the beginning of our treatment, to wash out with the syringe as much of the offensive matters as possible, and in the first wipings care must be taken not to employ enough cotton upon the broach to make it fit the canal tight, for if we make a piston of it we force foul gas and offensive matters through the apex of the tooth, poisoning the alveolas periosteum and pericementum. Often the greatest care does not suffice to prevent this, especially in old cases where the apicial foramen has become enlarged by absorption. It is usually necessary to treat these cases for ten days or more, repeatedly washing and wiping until absolute cleanliness is obtained. We generally employ alcohol at the last, and we know the canal is clean when the cotton is withdrawn unstained, and odorless. As soon as the discharge of pus from the canal ceases we begin to take care about the crowding of foreign substances into the tooth, and never dismiss the patient without packing a little cotton saturated with alcohol into the canal. This can be easily caught upon a nerve-broach and withdrawn upon his return. When satisfied of absolute cleanliness it is our custom to make a tentative stopping of the tooth by filling the major part of lhe canal with cotton and alcohol, closing the orifice with gutta-percha. This we leave perhaps a month, when, if the case has progressed favorably and all inflammation has subsided, we proceed to fill the canal permanently.—*Dental Practitioner.*

